# EHMTI-0044. Variability of clinical features in attacks of migraine with aura

**DOI:** 10.1186/1129-2377-15-S1-D41

**Published:** 2014-09-18

**Authors:** J Møller Hansen, PJ Goadsby, A Charles

**Affiliations:** 1 Department of Neurology: Headache Research and Treatment Program, University of California Los Angeles, Los Angeles, USA; 2 Department of Neurology: Headache Group, University of California San Francisco, San Francisco, USA

## Background

There is significant variability in the clinical presentation of migraine, among patients, and between attacks in an individual patient. We examined clinical features of migraine with aura in a large group of patients, and compared retrospective and prospective patient reports of migraine attack characteristics.

## Methods

267 patients with migraine with typical visual aura provided a detailed retrospective description of the clinical features of their attacks of migraine. During the study, clinical symptoms in migraine attacks starting with aura were recorded prospectively in 861 attacks.

## Results

Retrospectively reported visual aura symptoms were variable and often overlapping; the most common symptoms were dots or flashing lights, wavy or jagged lines, blind spots, and tunnel vision. Non-visual aura symptoms were reported by approximately half of the patients, the most prevalent being numbness and tingling, followed by difficulty in recalling or speaking words. There were significant inconsistencies between the features of prospectively and retrospectively recorded attacks. Headache, nausea, photophobia, and phonophobia were all less common in prospectively recorded attacks as compared with retrospective reporting. Nausea was prospectively reported in only 51% of attacks and mostly with mild intensity. The occurrence and severity of nausea was reduced with advancing patient age. Phonophobia was not consistently reported in conjunction with photophobia.

## Conclusion

These findings indicate variable involvement of different brain regions during migraine attacks. The variable occurrence of nausea, and of phonophobia in conjunction with photophobia may be an important factor in clinical studies where attacks are diagnosed based on these clinical features.

No conflict of interest.

